# Extracellular milieu grossly alters pathogen-specific immune response of mammary epithelial cells

**DOI:** 10.1186/s12917-015-0489-3

**Published:** 2015-07-30

**Authors:** Isabel Bauer, Juliane Günther, Thomas T. Wheeler, Susanne Engelmann, Hans-Martin Seyfert

**Affiliations:** Institute for Genome Biology, Leibniz Institute for Farm Animal Biology, Wilhelm-Stahl-Allee 2, 18196 Dummerstorf, Germany; Dairy Foods, AgResearch Ltd, Ruakura Research Centre, Hamilton, 3240 New Zealand; Institute for Microbiology, Technical University of Braunschweig, Spielmannstraße 7, 38106 Braunschweig, Germany; Helmholtz Center for Infection Research, Microbial Proteomics, Inhoffenstraße 7, 38124 Braunschweig, Germany

**Keywords:** Cattle, *Escherichia coli*, Inflammation, Innate immune response, Mastitis, NF-κB, Serum, *Staphylococcus aureus*, TLR signalling

## Abstract

**Background:**

Considerably divergent data have been published from attempts to model the *E. coli* vs. *S. aureus* specific immune reaction of the udder using primary cultures of bovine mammary epithelial cells from cows (pbMEC). Some groups reported a swift, strong and transient inflammatory response against challenges with *E. coli* and only a weak and retarded response against *S. aureus*, in agreement with the respective reaction of the udder. Others found almost the reverse. Presence or absence of fetal calf serum distinguished the experimental setting between both groups. We examined here if this causes the divergent reaction of the pbMEC towards both pathogen species. We challenged pbMEC with proteins from heat killed *E. coli* or *S. aureus* pathogens or purified TLR2 and TLR4 ligands. The stimuli were applied in normal growth medium with (SM10) or without (SM0) 10 % fetal calf serum, or in the basal medium supplemented with 10 mg/ml milk proteins (SM Milk).

**Results:**

Withdrawal of FCS slowed down and decreased the extent by which *E. coli* or LPS enhanced the expression of cyto- and chemokine encoding genes through impaired TLR4 signalling but enforced their expression during stimulation with *S. aureus*. SM Milk strongly quenched the induction of those genes. *S. aureus* strain specific differences in the reaction of the pbMEC could only be recorded in SM0. NF-κB factors were activated by *E. coli* in all stimulation media, but only to a small extent by *S. aureus,* solely in SM0. Purified ligands for TLR2 stimulated expression of those genes and activated NF-κB equally well in SM10 and SM0. The mRNA destabilizing factor tristetraproline was only induced by *E. coli* in SM10 and by purified PAMPs.

**Conclusions:**

Our data cross validate the correctness of previously published divergent data on the pathogen-specific induction of key immune genes in pbMEC. The differences are due to the presence of FCS, modulating signalling through TLR4 and TLR-unrelated pathogen receptors. *S. aureus* does not substantially activate any TLR signalling in MEC. Rather, receptors distinct from TLRs perceive the presence of *S. aureus* and control the immune response against this pathogen in MEC.

**Electronic supplementary material:**

The online version of this article (doi:10.1186/s12917-015-0489-3) contains supplementary material, which is available to authorized users.

## Background

Infections of the udder (mastitis) are a key disease for dairy animals [1]. The course of the infection and its resolution depend heavily on the species of the invading pathogen [2, 3]. *Escherichia coli* (*E. coli*) and other Gram-negative bacteria very often cause heavy inflammation of the udder and severe clinical symptoms (acute mastitis). The vigorous immune response towards those pathogens is driven by the massive, but transient synthesis and secretion of master cytokines (for example tumor necrosis factor α; interleukin 1 β [TNF; IL1B]) in the udder [4]). These infections are often self-healing by eradicating the pathogens. *Staphylococcus aureus* (*S. aureus*) and other Gram-positive germs however tend to elicit only mild inflammations (subclinical mastitis) due to belated and diminished induction of cytokine synthesis. These infections may become persistent and chronic [5].

Primary cultures of bovine mammary epithelial cells (pbMEC) have been used in a variety of studies as models for analysing the molecular causes underpinning the pathogen-species dependence of the immune response in the udder. We and others found consistently that pbMEC properly recapitulate the pathogen specific differences recorded from experimentally infected cows. They responded quickly after a challenge through heat inactivated *E. coli* pathogens or lipopolysaccharide (LPS) with a strong induction of key immune genes. This was always transient for most of the cytokine- and chemokine-encoding genes. The response towards similar preparations of *S. aureus* or lipoteichioc acid (LTA) was always belated and much weaker [6–12]. Pathogen-specific differentiated induction of the nuclear factor kappa-light-chain-enhancer of activated B cells (NF-κB) factor complex [13] in the pbMEC was identified as a major cause for these qualitative and quantitative differences in the immune response. Challenging the pbMEC with heat killed *E. coli*, but not with *S. aureus* activated the NF-κB factor complex, albeit that both pathogens would trigger toll-like receptor 2 (TLR2) or TLR4 mediated NF-κB activation in other cell types, such as HEK293 cells [9].

However, other groups using in principle the same cell model found that *S. aureus* would elicit an immune response as quickly as – or even faster than – *E. coli* of eventually almost the same intensity [14–17]. Moreover, the *E. coli* stimulus did not cause a peak-shaped and transient induction of cytokine gene expression but rather resulted in a sustained induction, notably of *TNF*, *IL1B* and of the chemokine *CXCL8* [14, 17]. These differences in modelling the pathogen–species specific immune response of the udder using pbMEC are to date unexplained. They raise doubts about eventually biased interpretations of key mechanisms governing the pathogen specific immune response in the udder.

A comparison of the methods applied in these studies revealed as a main difference the addition of 10 % fetal calf serum (FCS) to the growth medium of pbMEC during the stimulation period. Those groups having found the fast and transient response towards *E. coli* and LPS always stimulated the pbMEC in the presence of 10 % FCS, while all the other studies reporting a strong and transient response towards *S. aureus* and a delayed response towards *E. coli* deprived the cell growth medium of FCS during the challenge.

Depriving cells from serum deeply impacts on cell metabolism, cell cycle progression and induces apoptosis [18–20]. Serum starvation is a widely used condition for the analysis of immune responsiveness of model cells. However, it has already been shown in the HEK293 reconstitution system of TLR4 signalling that the LPS mediated TLR4 activation is dampened and retarded in FCS free stimulation medium due to the concomitant lack of LPS binding protein (LBP) [21]. Moreover, it was found in murine RAW264 macrophages that the addition of FCS accelerated the TLR4 mediated response to LPS [22]. The quality of FCS was identified as a key variable in the pathogen dependent interferon γ induction of human T-cells [23]. Withdrawal of FCS was shown to massively activate the basal level of NF-κB factors in COS cells, suggesting the existence of an inhibitor of NF-κB activation in fetal calf serum, acting through G-protein coupled receptors [24]. Base line expression of the master cytokine interleukin 6 (IL6) was found to be strongly increased by supplementing the growth medium of bone marrow cells with FCS [25]. Based on qualitative Western blot data, a recent report suggests that some *S. aureus* mediated NF-κB activation may occur in pbMEC stimulated in serum free, but not in serum containing medium [26]. These different lines of evidence all suggest that serum starvation might impact on the recognition of different pathogen species by the host cell. Hence, the composition of the growth and stimulation medium might crucially influence key mechanisms of immune induction in the MEC.

We therefore analysed here the influence of FCS upon the pathogen-species specific immune response of pbMEC. In addition, we evaluated the effect of milk proteins upon pbMEC activation, since MEC are *in vivo* exposed to milk. We found that the presence of 10 % FCS is necessary for a swift and strong induction of the expression of cytokine and chemokine encoding genes in pbMEC after stimulation with *E. coli*, while significantly attenuating the response towards *S. aureus.* Addition of milk constituents quenched the response of these cells towards both pathogens, but strongest towards *S. aureus*.

## Results

### Replica experiments yielded in principle concordant results but with slightly different kinetics

We monitored the transcript levels of genes encoding the immediate early immune response factors (cytokines and chemokines; Group A genes) TNF, IL1A, IL1B, IL6, CXCL8 (also known as IL8) and CCL20 to analyse the early pathogen specific response in primary cultures of bovine MEC (pbMEC). Transcript levels of genes encoding the inducible nitric oxide synthase (NOS2A), β defensin LAP and serum amyloid A3 (SAA3) served as markers for the secondary, late pathogen specific response and we are referring to them as Group B genes. Transcript levels from the gene encoding tristetraprolin (TTP) served as a control for the activation of mRNA decay [27].

The data set to evaluate the effect of different compositions of the stimulation medium upon the pathogen-specific immune response was derived from biological replica experiments (each assayed in duplicate) using pbMEC obtained from at least two different cows. The pbMEC were routinely grown in RPMI 1640 medium supplemented with 10 % FCS (Stimulation Medium SM10). Stimulation of pbMEC was carried out by either adding 30 μg/ml of the heat inactivated pathogen particles (*E. coli* strain 1303; *S. aureus* strain 1027 [28]) directly to cells grown in SM10 or after replacing the growth medium by RPMI 1640 without FCS (= SM0), or RPMI 1640 (without FCS) supplemented with 10 mg/ml of milk proteins (= SM milk). The concentration of bacterial proteins added represents approximately 10^7^ pathogen particles per ml (MOI of ~30). Applying this challenge dose of both pathogens in SM10 resulted in a strong activation of the bovine TLR2 receptor in the HEK293 reconstitution system of TLR-signalling (Additional file 1: Figure S1A), similarly as previously reported [9].

The induction kinetics of TNF-encoding mRNA levels exemplified the qualitatively good agreement of replica experiments (Additional file 1: Figure S2). The kinetics revealed that the response was somewhat faster and more intense in experiment 2. *E. coli* increased the *TNF* concentration in SM10 by 153 fold in experiment 1 and by 245 fold in experiment 2. Similarly, the *TNF* concentration was increased in response to *S. aureus* by 14 fold in SM0 in experiment 1 and by 87 fold in experiment 2. Differences in induction rates and kinetics resulted in a considerable variance of mean values for relative mRNA levels in both experiments. However, the following differences of the pathogen-specific induction of the candidate genes were concordantly found in replica experiments.

### Withdrawal of FCS quenched and retarded the *E. coli* induced expression of group A genes

**Fig. 1:**
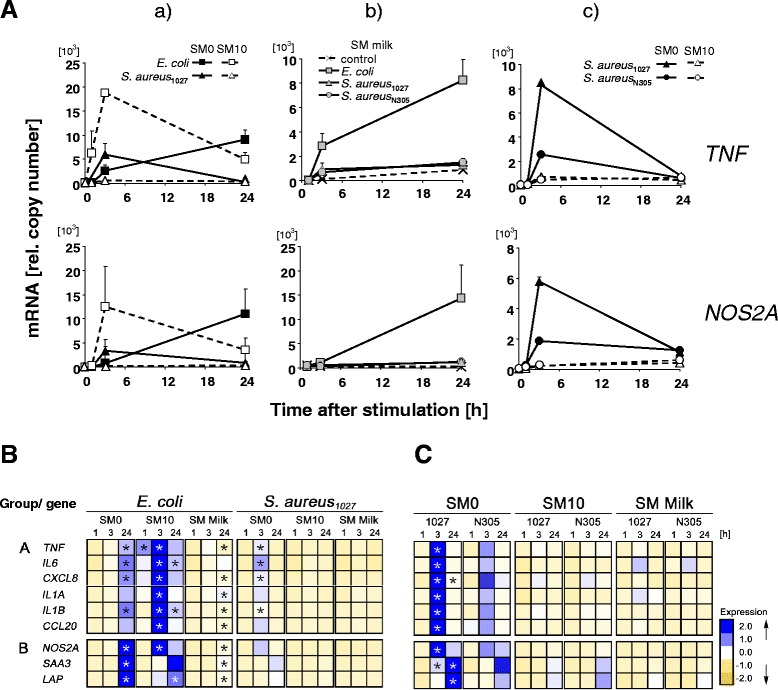
Composition of the challenge medium influences extent and kinetic of pathogen-specific induction of *TNF* and *NOS2A* expression. **a** *Influence of the different stimulation media upon extent and kinetics of the pathogen specific induction of TNF and NOS2A expression in pbMEC. a*) Effect of absence (SM0) or presence (SM10) of 10 % FCS in the challenge medium to which 30 μg/ml of heat killed *E.coli*_1303_ or *S. aureus*_1027_ had been added. The mRNA concentration (ordinate) was measured at different times after challenge (abscissa). *b*) Same as *a*), but the challenge medium was supplemented with 10 mg/ml of total milk protein (SM milk) and *S. aureus* strain N305 was also included. Note that the addition milk increased the base line expression (control). *c*) Same as *a*), but the challenge was done with two *S. aureus* strains. Values are means and SEM. (error bars) from two independent biological replica, each sampled in duplicate. **b** Data are from the same experiments as shown in (**a**), but the EXPANDER software was used to display the data for several genes. Each line displays the fold changes (compared to the unstimulated control) in the expression of the respective gene as indicated over the time of the challenge (1, 3, 24 [h]), normalized across all conditions to the average of 0 and variance 1. Data have been taken from Additional file 2: Table S1A. Group A, immediate early genes, Group B, secondary immune response genes. **c** Same as (**b**), but the analysis included only the data from the two different S. aureus strains (indicated). Data have been taken from Additional file 2: Table S1B *, *p* < 0.05 vs. unstimulated control (t-test, with Bonferroni’s correction)

Withdrawal of FCS significantly belated the *E. coli* induced expression of immediate early genes. For example, challenging with *E. coli* in SM10 caused swiftly a steep (189 ± 4.4 fold, mean ± SEM; n, 2) peak shaped increase in the *TNF* concentration already three hours after the challenge. In contrast, applying the same challenge in SM0 caused a slower, yet steady increase of that mRNA concentration, resulting in a 50.2 ± 6.5 fold increase at 24 h after the challenge (Fig. 1aa; Additional file 2: Table S1A). Similarly, the maximum mRNA concentration of all other Group A genes was reached 3 h after challenging in SM10 and then declined down to approximately 20 % of that peak value at 24 h post challenge (Fig. 1b; Additional file 2: Table S1A). In contrast, all these genes achieved their maximum mRNA concentration not earlier than 24 h after stimulation with *E. coli* in SM0.

Withdrawal of FCS reduced the extent of *E. coli* induced expression, particularly of Group A genes. The maximum mRNA concentrations of these genes after *E. coli* stimulation in SM0 was on average only about 1/3^rd^ of that recorded in SM10 (Fig. 1b).

The FCS related influence upon the *E. coli* induced expression of the secondary response Group B genes was not so strong, neither regarding kinetic nor extent of the modulation.

### Withdrawal of FCS strongly enhanced extent, but not the kinetic of *S. aureus* induced immune genes expression

Stimulation with *S. aureus* in SM0 strongly increased the maximum mRNA concentration of Group A genes (Fig. 1aa; b), on average more than fivefold. This was similar to the average of the quantitative effect upon Group B gene expression.

The composition of the stimulation medium did not alter the time course of gene induction during stimulation with *S. aureus*. Maximum mRNA concentrations of immediate early (Group A) genes were always reached three hours after stimulation, irrespective of the presence of FCS in the stimulation medium. However, only in SM0 was their peak shaped induction very obvious, due to their poor quantitative induction in SM10.

These data together show that presence or absence of 10 % FCS in the stimulation medium multiplied quantitatively into an almost twenty fold difference in the extent of the *E. coli* vs. *S. aureus* specific induction of the expression of cyto- and chemokine encoding genes.

### Milk proteins generally reduced the pathogen-related induction of almost all genes

Milk samples taken from an individual cow reflect the actual condition inside the udder at the time of sampling. These conditions might be very variable**.** Moreover, we realized previously in a study unrelated to the present context, that a short temperature treatment (72 °C, 15 s) known as pasteurization grossly alters the immune stimulatory properties of milk. We found that raw milk, but not pasteurized milk significantly increased the base line expression of immune genes, such as TNFα or LAP (Additional file 1: Figure S5A, B). Hence, the use of cow’s milk is difficult to standardize. In addition, it proved to be a lengthy process to legally import lyophilisates of those milk samples from healthy cows across the borders of countries. We therefore used for the study here a commercially worldwide available milk powder as a supplement for the stimulation medium.

Initial experiments showed that addition of milk powder, re-dissolved in RPMI 1640 diminished the pathogen-induced response of all indicator genes in a dose dependent fashion and also increased the baseline transcription of several Group A genes in non-challenged control cultures (data not shown). We therefore stimulated the cells in RPMI 1640 to which 10 mg/ml of re-dissolved milk proteins had been added (SM Milk) and recorded the base line expression from control cultures at any time point.

Addition of milk proteins consistently increased the basal transcript level of the marker genes over time but with different intensities for individual genes. For example, the highest increase was found for *IL1B* (53.9 ± 15.9 fold, mean ± SEM from two biological replica experiments) and *SAA3* (42.2 ± 1.0 fold) while for *IL1A* only a 2.5 ± 0.1 fold increase was detected (Additional file 1: Figure S3). For comparison, we found that just removing the FCS from the medium (e.g. maintaining the cells for 24 h in SM0) had a weaker effect upon the base line expression of those genes, with the noteworthy exception that removal of serum reduced the base line expression of CXCL8, IL1A and IL6. The base line expression of all genes remained stable in SM10 over the entire 24 h of the experiment in SM10.

That strong effect of milk over time on the base line expression of cytokine encoding genes resulted in a diminished proportion of the pathogen related increase in the mRNA concentrations in SM Milk (Additional file 2: Table S1A). Stimulation with *E. coli* in SM Milk resulted in a similar stimulation pattern of immune gene expression as recorded in SM0 (Fig. 1aa vs. ab). This refers to both, extent and kinetic of increased mRNA abundance post challenge. In contrast, stimulation with *E. coli* in SM10 or SM Milk differentially modulated for several genes the kinetic of their induction. Key difference in this regard was in SM Milk the lack or only marginal down regulation (e.g. degradation) of the mRNAs, similar as seen in SM0.

Challenging the pbMEC with *S. aureus* in SM Milk did not alter the kinetic of modulated gene expression compared to *S. aureus* stimulation in both other media (Fig. 1a*b*). It resulted in the same very weak increase in the mRNA concentration of Group A and Group B genes as recorded after stimulating with S. aureus in SM10 (Additional file 2: Table S1A).

### *S. aureus* strain specific differences in the capacity to induce immune gene expression in pbMEC can only be recorded in SM0

A previous study reported that different *S. aureus* isolates from cases of mastitis differentially induced immune gene expression in pbMEC [14]. This study had been conducted stimulating with live bacteria for three hours and using a FCS depleted stimulation medium (comparable to SM0). Unexpectedly, we could subsequently not find any statistically significant difference in the immune induction capacities of various *S. aureus* isolates from cases of bovine mastitis (Additional file 1: Figure S4). These isolates had previously been shown to be genetically quite diverse [29]. However, our studies had been conducted using SM10. We had already seen in pilot experiments that co-culturing live *S. aureus*_1027_ with pbMEC in SM10 for 1 h did not improve the stimulatory properties of this strain, neither regarding the induced expression of immune genes nor the activation of NF-κB (Additional file 1: Figure S4). Hence, it was unlikely that incubation with heat killed rather than live *S. aureus* pathogens would be causative for our always recorded uniform low capacity of *S. aureus* to induce immune gene expression in pbMEC. We therefore compared now in some experiments properties of the quite commonly used *S. aureus* strain Newbould 305 (N305 [14] to those of our ‘standard’ *S. aureus* strain 1027. We observed statistically significant differences between both strains only after stimulation in SM0, but neither in SM Milk nor in SM10 (Fig. 1ab, ac; c). Stimulation with *S. aureus* 1027 in SM0 increased the mRNA concentration of all group A and B genes more than 3 fold over the level as induced by N305 (Table 1). In contrast, the induction capacity of both strains was virtually the same in SM10 or in SM Milk.

**Table 1:** Comparison of the induction capacity of *S. aureus* strain 1027 vs. N305 in different stimulation media

Group	Gene	SM0	SM10	SM milk
A	TNF	3.33^a^ ± 0.08	1.33 ± 0.23	0.88 ± 0.03
	IL6	3.85 ± 0.27	1.09 ± 0.08	1.16 ± 0.31
	IL1A	2.17 ± 0.77	1.02 ± 0.17	1.45 ± 0.25
	IL1B	4.17 ± 0.80	1.18 ± 0.01	0.68 ± 0.06
	CXCL8	2.17 ± 0.25	1.06 ± 0.11	1.64 ± 0.20
	CCL20	4.76 ± 0.95	0.75 ± 0.05	1.06 ± 0.12
B	NOS2A	3.70 ± 0.53	1.10 ± 0.11	0.99 ± 0.07
	LAP	2.78 ± 0.25	1.02 ± 0.38	0.89 ± 0.03
	SAA3	1.72 ± 0.07	1.09 ± 0.42	0.79 ± 0.13

^a^Ratio of the maximum fold change induced by strain 1027 vs. N305; mean value ± SEM form two biological replica, each assayed in duplicate

### *E. coli* activated NF-κB in all media, but *S. aureus* only marginally in SM0

**Fig. 2:**
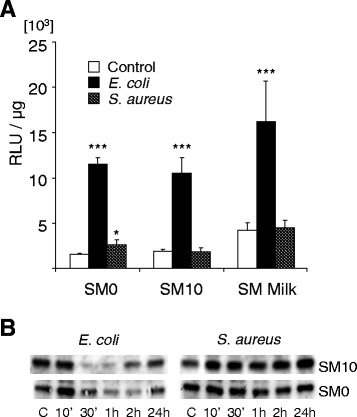
Influence of medium composition on pathogen or PAMP mediated NF-κB activation in pbMEC. **a** E. coli*, but not* S. aureus *activates NF-κB in all media*. pbMEC were transiently transfected with the NF-κB driven luciferase reporter gene and subsequently challenged for 24 h with 30 μg/ of heat killed pathogens in the media as indicated and the luciferase activity was measured from lysates (ordinate). Mean values (± S.E.M.) from five experiments (four biological replica) for SM0 and SM10, and two biological replica experiments for SM Milk. Each experiment was assayed in triplicate (*, *p* < 0.05; ***, *p* < 0.001 vs. control). **b** *Only the E. coli challenge degrades IκBα*. Degradation of IκBα was visualized in Western-blots after challenging the pbMEC in SM0 or SM10 with 30 μg/ml of heat killed *E. coli* or *S. aureus*_1027_ particles. Thirty μg of cell lysates having been collected from unstimulated control cultures (c) or at the times as indicated after the challenge had been resolved in any slot of a 12 % SDS-gel and blotted onto nitrocellulose. The primary antibody was specific for IκBα. Data are representative for two independent experiments

We analysed the influence of the different stimulation media upon the activation of the NF-κB factor complex in order to search for mechanisms underpinning the improved induction of immune gene expression by *S. aureus* in SM0. NF-κB activation is an integrating parameter for TLR-signalling. We therefore transfected a NF-κB transcription factor driven luciferase expressing reporter gene into pbMEC and found that *E. coli* activated NF-κB factors ~5 fold in all three media while *S. aureus* 1027 did so only in SM0 and only to a small extent (Fig. 2a). *S. aureus* activated the level of active NF-κB factors by 1.70 ± 0.08 fold in SM0 (five separate experiments including pbMEC derived from four different cows). Analysis of the NF-κB inducing capacity in pbMEC of four other *S. aureus* strains in SM10 revealed that they all failed to activate NF-κB factors in the FCS containing growth medium SM10. Even co-culturing for 1 h live *S. aureus*_1027_ pathogens with the pbMEC in SM10 did not significantly induce the level of active NF-κB factors (Additional file 1: Figure S4). Addition of milk proteins allowed for a highly significant NF-κB activation after stimulation with *E. coli*, but virtually no induction after stimulation with *S. aureus*_1027_. Addition of milk proteins significantly increased the basal activity of NF-κB factors in the unstimulated control cultures during the 24 h incubation period by 2.8 fold (*p* < 0.01; n, 2, each assayed in triplicate).

We analysed in Western blots on a time line (10 min–24 h) the influence of FCS upon *S. aureus* induced degradation of the NF-κB inhibitor protein IκBα serving as a second parameter for NF-κB activation. Stimulation with *E. coli* resulted in a degradation of IκBα in both media, SM10 and SM0, while this was not apparent after stimulation with *S. aureus* (Fig. 2b). The *E. coli* challenge in SM10 resulted already after 30 min in a very clearly visible degradation of the IκBα factor, and its re-synthesis was apparent after 2 h. This process was retarded after stimulation in SM0, complying well with the delayed induction of the expression of our indicator genes in SM0. In contrast, IκBα was not degraded by *S. aureus* stimulation, neither if applied in SM0 nor in SM10. Interestingly, neither of the pathogens degraded this factor in SM Milk (not shown).

### FCS modulated the effect of TLR4-, but not of TLR2- mediated signalling

**Fig. 3:**
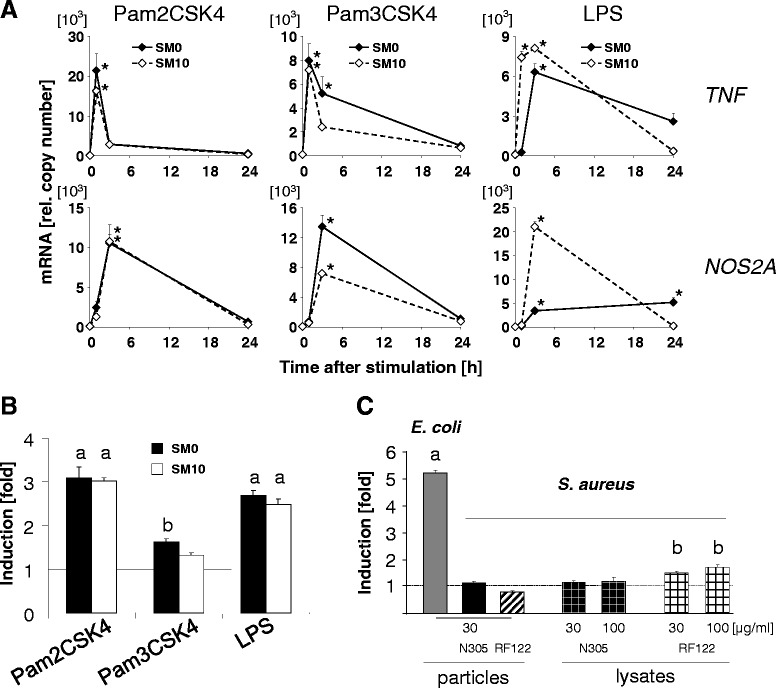
Dissection of the influence of FCS upon TLR2 or TLR4 mediated pathogen recognition. **a** TLR2, but not TLR4 signalling activates immune gene expression irrespective of FCS presence. pbMEC were challenges in SM10 or SM0 with synthetic lipopetides (Pam2CSK4, 10 ng/ml; Pam3CSK4, 100 ng/ml) as TLR2 ligands or purified LPS (10 ng/ml) as TLR4 ligand. Same experimental setting as in Fig. 1. The mRNA concentration (ordinate) of *TNF* or *NOS2A* was measured at different times after challenge (abscissa). Data are mean values and SEM from two biological replica experiments, each assayed in duplicate; *, *p* < 0.05 vs. unstimulated control (t-test, with Bonferroni’s correction). **b** Purified PAMPS activate NF-κB in pbMEC irrespective of FCS supplementation. pbMEC were transfected with the NF-κB reporter expressing vector (same experimental setting as in Fig. 2a) and challenged for 24 h with purified LPS or Pam2CSK4 (10 ng/ml each) or 100 ng/ml of Pam3CSK4. Mean values of increased luciferase activity over that of unstimulated controls (ordinate) from two (LPS, Pam2CSK4) independent experiments, each assayed in triplicate. Different superscript letters indicate significant (t-test, *p* < 0.05) difference from each other and from the unstimulated control. **c** Lysates from shattered S. aureus do not strongly activate NF-κB in pbMEC. Same as **b**, but comparing in pbMEC the NF-κB activation through heat killed bacteria (particles) with that caused by supernatants (lysates) from RiboLyser crushed heat killed pathogens from *S. aureus* strains N305 and RF122 to that of *E. coli*. Protein concentrations applied for the respective challenge are given below the columns. Each condition was assayed in triplicate. Superscript letters (a, b) indicate significant (*p* < 0.05) difference between the column means and from the unstimulated controls

We wondered if the impaired immune stimulation of our pbMEC cells by *S. aureus* applied in SM10 might be due to FCS-mediated impaired ligand recognition by the TLR2 receptor. The synthetic lipopeptides Pam2CSK4 and Pam3CSK4 are widely used as mimetic for bacterial di- or tri-acetylated lipoproteins. They are ligands for TLR2/6 and TLR2/1 heterodimers, respectively [30]. It was previously shown that Pam2CSK4 is a much more potent activator of CXCL8 expression in pbMEC than Pam3CSK4 [31]. We compared the efficacy of both lipopeptides to stimulate the TLR2 receptor in the HEK293 cells (Additional file 1: Figure S1B) in order to find physiologically comparable dosages. Based on these data we stimulated the pbMEC cultures in SM0 and SM10 with 10 ng/ml of Pam2CSK4 and 100 ng/ml of Pam3CSK4 and included a highly purified LPS preparation from *E. coli* 1303 as a pure TLR4 ligand [9]. Both model substances for lipopeptides stimulated very strongly and quickly the expression of *TNF* in both stimulation media (Fig. 3a) similarly as it was found for other Group A genes (Additional file 2: Table S1B). Stimulation with Pam2CSK4 increased the maximum *TNF* concentration to approximately the level as found after stimulation with *E. coli* in SM10. The same applied to the NOS2A mRNA concentration. LPS, on the other hand stimulated the expression faster and stronger in SM10 than in SM0.

All three purified PAMPs (both lipopeptides, LPS) activated NF-κB factors in pbMEC irrespective of the addition of FCS (Fig. 3b).

These data together reveal proper, ligand dependent signalling of TLR2 in MEC irrespective of the presence of FCS. They also show that the TLR4 receptor was activated by its specific ligand irrespective of FCS presence, but that physiological consequences of TLR4 activation (e.g. activation of immune genes expression) were indeed modulated by FCS.

### Mechanically shattering *S. aureus* enhanced their immune stimulatory efficacy in SM10

Impaired immune stimulatory efficacy of the *S. aureus* particles in SM10 but proper response of the cells towards purified mimetics of authentic bacterial PAMPs suggested that these might not be adequately recognisable by pbMEC, if structurally still arranged in the cell wall of the bacterium and administered in SM10. Hence, we used a RiboLyser device to brake-up and shatter *S. aureus* in order to possibly excavate more of these PAMPs. We challenged the pbMEC with particle free supernatants of such lysates for three hours in SM10. We found for two different *S. aureus* strains that this treatment indeed significantly enhanced their immune stimulatory properties towards pbMEC over the level recorded with heat killed pathogens from these strains (Table 2). The average enhancement of pathogen induced expression of six immune genes was 5 to >11 fold (for strains RF122 and N305, respectively) and expression of the NOS2A gene was enhanced by spectacular 67 to >150 fold by disrupting the pathogens in the RiboLyser. However, these lysates of the shattered *S. aureus* only marginally activated NF-κB in SM10 (Fig. 3c).

**Table 2:** Enhanced immune gene induction by *S. aureus* in SM10 through pathogen disruption

Gene	N305	RF122
CXCL8	5.6	6.5
TNF	14. 9	5.9
IL1B	25.5	6.7
IL1A	4.2	4.0
IL6	9.4	5.4
SAA3	8.3	5.5
NOS2A	159.4	67.3

Values are the ratio of increased mRNA concentration (fold over control) after 3 h stimulation with 30 μg/ml of RiboLyser lysates and the corresponding value obtained after stimulating with 30 μg/ml of the respective pathogen particles. Induction values for particles and RiboLyser extracts had been obtained in separate experiments, each strain assayed in duplicate

### Differential influence of FCS upon TLR ligand-specific induction of tristetraproline expression

**Fig. 4:**
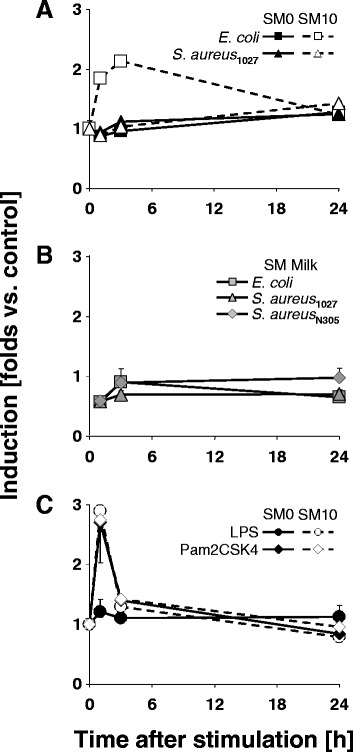
Modulation of TTP expression through FCS. **a** TTP expression is only activated by E. coli particles in SM10. pbMEC were stimulated with 30 μg/ml of heat killed pathogens media with or without 10 % FCS (SM10 or SM0), for the time as indicated. The fold increase in the TTP mRNA concentration above the control value is indicated. Mean values ± SEM from two biological replica experiments, each assayed in duplicate. Only values from *E. coli* stimulation at 1 h and 3 h in SM10 resulted in significantly increased mean values (*p* < 0.001). **b** Addition of milk prevents induction of TTP expression. Same experimental setting as above, but stimulation was in SM Milk. No significant induction of TTP mRNA concentration, but a significant (*p* < 0.05) decrease compared to the unstimulated control at t 1 h. **c** Pam2CSK4, but not LPS triggers TTP expression irrespective of the presence of FCS. Same experimental setting as in A) but stimulation was with 10 ng/ml of either Pam2CSK4 or highly purified LPS. All values, but that for LPS at 1 h after the challenge differed significantly (*p* < 0.01) from the control value

Tristetraproline (also known as TTP, encoded by the ZFP36 gene) is known to bind to the AU-rich destabilizing ARE-sequence elements in the 3′-UTR regions of cytokine and chemokine-encoding mRNAs and plays a role in their active degradation. We have previously reported that the mRNA concentration of this gene as well as the abundance of the encoded enzyme is increased after challenging pbMEC with *E. coli* in SM10 [7]. We therefore examined if eventually a media differentiated induction of this factor might contribute to modulate the down regulation of the cyto- and chemokine-encoding (Group A) gene expression after *E. coli* stimulation. Considering the pathogen particles we found that only challenging the pbMEC in SM10 with *E. coli* resulted in a significant induction of the mRNA concentration of this gene (Fig. 4). Its expression was neither induced by *E. coli* in the two other media nor by *S. aureus* in any of the stimulation media. Yet, Pam2CSK4 (similarly Pam3CSK4, not shown) activated TTP expression in SM0 as well as in SM10 to a similar extent as recorded after stimulation with *E. coli* in SM10. LPS, on the other hand induced TTP expression only in SM10, but not in SM0.

## Discussion

The analysis of molecular mechanisms underpinning the pathogen-specific resolution of mastitis eventually requires using relevant model cells in adequate conditions. It is widely accepted that primary cultures of mammary epithelial cells may serve as relevant cell models in this regard and some of its limitations have already been published [12]. We examined here the influence of FCS and milk proteins in the culture medium upon the pathogen specific immune response of pbMEC in order to better define those adequate conditions.

We found as a key observation that withdrawal of FCS during the stimulation of pbMEC with pathogens profoundly alters both, extent and kinetic of the pathogen dependent expression of immune genes. The effect of FCS alters more profoundly the pathogen specific reaction norm of the pbMEC than challenging them with either live or heat killed pathogens. The data of our study regarding extent and kinetics of pathogen-specific immune gene induction recorded in both stimulation media, with (SM10) and without (SM0) FCS fully comply with previously published data from stimulation of pbMEC with heat killed or live pathogens in the respective media. We observed that presence or absence of 10 % FCS introduces a very large difference in the extent by which *E. coli* vs. *S. aureus* induced in particular the cyto- and chemokine encoding immediate early immune genes. This readily explains why different groups have previously reported divergent data about both extent and kinetics of their induction by *E. coli* and *S. aureus* [6, 8, 9, 14, 17]. Moreover, the finding that the *S. aureus* strain specific influence can only be recorded in SM0 explains why we have so far been unable to detect such strain specific differences, albeit considerable efforts and using genetically diverse strains. The comparison made here between *S. aureus* strains 1027 and Newbold N305 conducted in serum free medium (SM0) complies with a previous report in the fact that N305 is a relatively weak inducer of immune gene expression in pbMEC [14]. We show here that the RiboLyser treatment enhanced its immune stimulatory properties and this to a larger extent than encountered with strain RF122. This implies that the heat killed particles of N305 did not expose all relevant immune stimulatory molecules to the pbMEC target cells. Moreover, the *S. aureus* strain specific differentiated effect of the RiboLyser treatment suggests that some of the strain specific properties may reside in the specific structural make-up of the bacterial cell wall.

By cross-validating the results obtained under the different experimental conditions our study may remove doubts about credibility of data and validity of interpretations of divergent data having been published by various groups during the last years.

### No recommendation can be given for an “adequate” stimulation medium

The nature of the *in vivo* environment of MEC is completely unknown. Neither are these cells in contact with a fluid containing 10 % of serum proteins (let alone fetal serum) nor in a fluid completely deprived of serum components and proteins. However, the apical membrane is in contact with milk, containing approximately 35 mg/ml of milk proteins. These include anti-microbial factors as well as auxiliary factors known to be highly relevant for pathogen recognition, such as LBP and CD14 [32–35].

Our study shows that one can pre-determine key features of the pathogen-species specific immune response of the pbMEC by selecting the specific stimulation condition. The choice of the medium should consider the parameter one seeks to analyse. Stimulating the pbMEC in serum containing medium (SM10) will result in a pathogen-species specific induction kinetic of immune genes just as one would expect to find based on the respective response of the udder *in vivo*, after infection of the gland with live pathogens. Stimulating them in SM0 with the same pathogen preparations will demonstrate a significantly induced expression of cytokine encoding genes by *S. aureus* or will allow to revealing *S. aureus* strain specific differences in the capacity to induce cytokine gene expression in pbMEC. Yet, one should avoid adding significant concentrations of milk proteins if the interest would be to explore the full capacity of the pbMEC to respond to an *S. aureus* stimulus with induced gene transcription. Moreover, the fact that not only the addition of serum protein, but also supplementation with milk-proteins eliminated any difference in the response of the MEC towards different *S. aureus* strains is thought-provoking about the physiological relevance of such differences having been revealed under serum- and milk-protein free stimulation conditions [14, 36].

Our results bear also on the molecular understanding of the pathogen-specific differentiated immune response of the MEC.

### Many serum effects upon the *E. coli* specific response are related to TLR4 signalling

Induced expression of the immediate early genes (cyto- and chemokine encoding genes) by *E. coli* and LPS was reduced and retarded in the FCS deprived SM0, similarly as the time course of NF-κB activation. Pathogen stimulus induced NF-κB activation in the target cell indicates TLR-mediated pathogen perception, since all TLRs are known to ultimately activate NF-κB factors. LPS is the dominant PAMP derived from *E. coli* and is the authentic ligand of TLR4 [37]. Full activation of TLR4 signalling through LPS requires that it is bound by the lipopolysaccharide binding protein (LBP) and that the auxiliary co-factors MD2 (also known as LY96) and CD14 contribute to present the ligand to the TLR4 receptor [21, 38–40]. LBP may be found in blood serum or milk [41], but is missing in plain RPMI 1640 medium. Thus, TLR4 mediated recognition of LPS may be impaired in SM0. It was found, indeed that withdrawal of FCS from the stimulation medium quantitatively reduced (but not abolished) TLR4 mediated NF-κB activation in HEK293 cells [21] and retarded IκBα degradation in RAW264 macrophages [22]. Our data in this regard agree excellently with these reports.

However, not all effects of serum withdrawal can unambiguously be attributed to impaired TLR4 signalling. The response of the pbMEC towards the *E. coli* stimulus was quite similar in SM0 and SM Milk, as was the extent of NF-κB activation after challenging these cells for 24 h (cf. Fig. 3a). The latter parameter indicates some TLR4 activation in all conditions, but the extent of increased cytokine mRNA abundance was six fold higher in SM10 at 3 h after stimulation than in both other media. Hence, FCS apparently provides some factors other than just LBP to both hasten and intensify expression of cytokine- and chemokine-encoding genes, but also that of NOS2A. Pleiotropic effects of FCS withdrawal were recently documented by showing that it not only attenuated and impaired TLR4 mediated NF-κB activation but also severely hindered the secretion of proinflammatory cytokines (TNF, CXCL10, for example) after LPS stimulation [42]. Thus, serum starvation influences post-transcriptional events which are so essential to eliciting a fully efficient immune response**.** The differential effect of FCS upon the expression of the mRNA destabilizing factor TTP is another example for multi-layered regulation through FCS (see below).

### *S. aureus* only very poorly activates NF-κB in pbMEC

Signalling from all TLR factors is known to ultimately converge in the activation of the NF-κB factor complex [43]. Hence, lacking NF-κB activation in SM10 or SM Milk and its only marginal activation in SM0 or through the RiboLyser extracts in SM10 together indicate missing or only low level of TLR activation through *S. aureus*. This low level of NF-κB activation was quantitatively equivalent to a previous report about challenging the established bovine MEC model cell MAC-T with heat killed *S. aureus* (1.3 fold induction in FCS containing medium, [44]). Assuming poor TLR activation by *S. aureus* is augmented by the obviously lacking degradation of the NF-κB inhibitor IκBα, a prerequisite for the classical route of TLR-mediated NF-κB activation [43, 45]. It might be that this method for monitoring NF-κB activation is just less sensitive than the reporter gene assay. Alternatively, the increased NF-κB activity picked-up in the reporter gene assay after *S. aureus* stimulation SM0 might have been caused other mechanisms activating NF-κB factors downstream of IκBα degradation. It is known that NF-κB factors (already liberated from the IκB complex) require additional modification through phosphorylation, for example [46, 47]. In this case mechanisms other than TLR-signalling might be responsible for the slight NF-κB activation recorded after stimulating the pbMEC with *S. aureus* in SM0. Anyhow, our data collectively show that *S. aureus* does not fully activate TLR signalling, in none of these media.

### TLR2 signalling is functional in MEC and not modulated by FCS supplementation

It is known from genetic analyses that perception of Gram-positive bacteria (such as *S. aureus*) crucially depends on the activation of TLR2 signalling, mainly conveyed through di-acetylated lipopeptides [48, 49]. We found in this regard that only the lipopeptide mimetics Pam2/3CSK4 strongly activated NF-κB factors in the pbMEC, hence activated TLR2 signalling. The effect can clearly be attributed to TLR2 signalling since the synthetic lipopeptides Pam2/3CSK4 have structurally been designed as specific TLR2 ligands. They activated with approximately equal efficacy in SM0 and SM10 both, NF-κB factors as well as the strong and rapid expression of cyto-and chemokine-encoding genes. Therefore, TLR2 signalling is fully functional in pbMEC and not compromised by FCS, provided that the PAMPs are offered as purified ligands. Similar observations have previously been published for different PAMPs (LTA, presumably activating TLR2 through co-purified lipopeptides [50]; muramyl dipeptides, activating NOD2 receptors) [51]). Hence, the failure of *S. aureus* to induce TLR-mediated NF-κB activation in pbMEC is not due to just obstructing the interaction of relevant PAMPs with TLRs (TLR2 in particular) through FCS. This conclusion is augmented by the strong TLR2 activation in the HEK293 reconstitution system of TLR signalling conducted in a 10 % FCS containing growth medium (DMEM; this study and [9]). It is more likely that PAMPs relevant for TLR2 activation are generally not recognizable by the TLR2 receptor if this is expressed in the MEC. The lipopeptides are apparently effectively hidden in the cell wall of *S. aureus.* The RiboLyser treatment did not liberate them efficiently enough to serve as functional TLR2 ligands.

### MEC perceive the presence of *S. aureus* independently from TLR-signalling

Induction of the expression of the immediate early genes by *S. aureus* appears to be independent from TLR signalling since this process, but not TLR2 signalling is strongly modulated by FCS. Expression of these genes is under multiple controls [52], including NOD2 receptors and MAP kinases, besides activation through the TLR-axis [43, 51]. Further support for assuming TLR-independent perception of *S. aureus* by MEC comes from the previous observation that challenging pbMEC in the presence of 5 % FCS with supernatants from *S. aureus* activates AP1 - rather than NF-κB - governed regulatory networks [53].

Our data regarding the influence of the medium composition for TLR signalling and *S. aureus* perception by the MEC collectively suggest that the TLR-axis has only a minor role for eliciting an immune alert against *S. aureus* in this cell type.

### Modulation of TTP expression exemplifies that the medium composition affects very different layers of immune gene regulation

Stimulation with *E. coli* in SM10 and *S. aureus* in SM0 both caused a peak shaped increase of the cyto- and chemokine –encoding genes followed in both conditions by a similar decline of the mRNA concentrations. Hence, we anticipated that also the expression of the mRNA decay factor TTP would be similarly triggered in both conditions. Yet, we found unexpectedly that the expression of this gene was induced by *E. coli* in SM10, but neither by *E. coli* in both other media, nor by *S. aureus* in any of these media. However, the pure ligands for TLR2 dependent signalling stimulated its expression irrespective of the presence of FCS. These data have various implications.

TTP expression was known to be under multiple controls and among them by serum factors and MAP kinases (see [54, 55] for a review). Our data add evidence that pure TLR2/4 ligands trigger the expression of this gene in pbMEC. The efficacy of the LPS challenge to induce TTP expression in SM10, but neither in SM0 nor in SM Milk supports by corollary the assumption that some facets of productive TLR4 signalling are active only in SM10, albeit activating NF-κB to a considerable extent in all three stimulation media. This shows that contact with milk or absence of FCS affects gene regulation in the MEC also at levels distinct from the TLR axis.

The medium dependent modulation of TTP expression complies well with the different kinetics of *E. coli* induced modulation of the mRNA levels of the immediate early genes in all three stimulation media. Particularly intriguing in this regard is the correlation of lacking TTP induction through *E. coli* in SM Milk and sustained high levels of the mRNA concentrations of Group A genes in this condition. However, our data imply as well that the down regulation of the mRNA concentrations of those Group A genes after stimulation with *S. aureus* in SM0 is mediated by mechanisms apparently being independent from induced TTP synthesis.

## Conclusions

We show here by cross-validating the correctness of previous reports that the choice of the culture fluid of pbMEC grossly alters the pathogen-species specific immune response of this cell type. Including FCS into the growth medium allows inducing in pbMEC an expression pattern of cyto- and chemokine encoding genes resembles phenotypically best what is known from the immune response of the udder during pathogen-species specific mastitis. Addition of fetal calf serum or milk proteins is essential for TLR4 mediated signalling enhancing the response towards *E. coli*. Omission of FCS will enforce *S. aureus* recognizing pathways independent from TLR-signalling. Our data generally confirm that *E. coli* but not *S. aureus* triggers strong TLR signalling in MEC in all conditions examined.

## Methods

### Chemicals and preparation of stimulation media

RPMI 1640 medium (Biochrom; Cat. No. F1215) was supplemented with prolactin, hydrocortisone, insulin as described [56]. Fetal calf serum (PAN-Biotech; 3302–251110) was from a lot which we had previously validated for not enhancing the base line expression of our candidate immune genes in pbMEC. Milk powder (Roth; T145.2) was of Western-blotting quality. The protein profile of this product was resolved in SDS-gels as a quality control and was found to be almost indistinguishable from that of cow’s milk (Additional file 1: Figure. S5). The powder was dissolved at a concentration of 35 mg/ml in basal RPMI 1640 medium (without fetal calf serum) by shaking overnight in the cold room (4 °C). The undissolved fines were spun down (14,000 × g; 30 min, 4 °C), the supernatant sterilized by filtration (0.2 μm filter) and adjusted to a concentration of 10 mg/ml by the addition of basal RPMI 1640 (SM Milk). Heat killed particles of *E. coli* strain 1303 and *S. aureus* strain 1027 were prepared as described [9]. LPS was prepared from strain *E. coli* 1303 essentially as described [11] and provided by Otto Holst (Forschungszentrum Borstel; Germany). We validated in the HEK293 reconstitution system of TLR signalling that this preparation activates the bovine TLR4, but not the TLR2 receptor. Synthetic lipopetides Pam2CSK4 and Pam3CSK4 were purchased from Invivogen.

### Disruption of *S. aureus* with the RiboLyser

*S. aureus* cell lysates were prepared as described previously [57]. Briefly, the *S. aureus* strains RF122 and N305 were grown in TSB medium to an optical density (OD540) of 7. Cells of 20 ml cultures were collected by centrifugation. Cell free supernatants were stored at − 20 °C. Cell pellets were washed with TE buffer, resuspended in TE buffer and transferred into screw top tubes containing 500 μl of glass beads. Cells were disrupted using the RiboLyser (Thermo Electron Cooperation) for 30 s at 6.5 m/s. Cell debris was removed by centrifugation and cell lysates were stored at − 20 °C.

### Experimental design

Cultures of pbMEC were established as previously described [56]**.** Cells were retrieved from healthy first lactating Holstein Friesian heifers having been slaughtered in our local abattoir, complying with all pertinent ethical and legal requirements. The abattoir is a EU licensed (ES1635) core facility of the research affiliation and serves to routinely supply samples to the different laboratories. Special ethical approval was unnecessary since the cows had been culled in the normal culling regime without conducting any animal experimentation. The cells were always grown to approximately 90 % confluence in collagen IV coated petri dishes (six well plates) in RPMI 1640 growth medium containing 10 % FCS. They were either stimulated in this medium (SM10) by the addition of 30 μg/ml of heat inactivated *E. coli* or *S. aureus* particles for various periods of time, or the cultures were washed twice with PBS and the culture medium was replaced by growth medium devoid of FCS (SM0) and the cells stimulated similarly as in SM10. Unstimulated cultures were kept as controls and were harvested after 24 h at the end of the experiment. The effect of milk was similarly evaluated, after replacing SM10 by SM Milk. However, separate unstimulated control cultures were kept in SM milk and harvested at any experimental time point. Each condition was evaluated from at least two biological replica experiments using pbMEC from different cows. Each stimulation experiment was assayed in duplicate.

The cells appeared to be healthy in all three media and under all diverse experimental conditions. This was not only clear from their appearance under the microscope, but also from consistently similar RNA yields under all conditions. We also checked in particular if *E. coli* or LPS stimulation would induce apoptosis using the apoptosis detection kit from Abcam (# ab14085) but did not find any evidence for this to occur under neither of the experimental conditions.

### RNA extraction and mRNA quantification

RNA extraction with TRIZOL-reagent (Invitrogen), cDNA preparation (Superscript, H^−^, Invitrogen) and real time quantification of the mRNA concentrations with the Fast-Start Syber Green I kit and the LightCycler II instrument (Roche) were done essentially as described [58]. Relative copy numbers were titrated against external standards consisting of dilution series of plasmids (10^6^ – 10 copies) harboring the respective amplicons. Values were normalized against the copy numbers of the not regulated reference gene chloride intracellular channel 1 (CLIC1). The virtually not regulated property of this gene emerged in our numerous whole transcriptome profiling studies of pbMEC [8, 11, 12]. We validated its stability by comparing the transcriptional response of CLIC1, RPL35A (ribosomal protein L35a), ACTB (β-actin) and GAPDH (glyceraldehyde 3-phosphate dehydrogenase) by challenging three biological replica of pbMEC from different animals with *E. coli* for 0, 1 and 24 h. The NormFinder software (http://moma.dk/normfinder-software) indicated the best stability for CLIC1 and the least for ACTB (NormFinder values 0.013 and 0.169 for CLIC1 and ACTB, respectively). The respective data and examples for the stability of CLIC1 expression under the various experimental conditions of this study are shown in Additional file 1: Figure S6).

Oligo nucleotide primers are listed in Additional file 2: Table S2.

### Determination of NF-κB activation

*Through TLR2 in the HEK293 reconstitution system of TLR signaling:* Validation of the activation of the bovine TLR2 receptor through its ligand specific NF-κB activation in the HEK293 system was conducted entirely as described [56]. Briefly, 100 ng of a vector expressing the TLR2 receptor from cattle were co-transfected with 40 ng of the NF-κB driven ELAM reporter gene construct (ELAM-promoter, Invivogen) into HEK293 into the cells of a single well from a 6-well plate. After recovering overnight, the cells were split and distributed into six wells of a 24 well plate. Three of these were stimulated for 24 h with the respective inducer and the other ones served as unstimulated controls. The luciferase activity was assayed from lysates with the dual luciferase assay reporter system (Promega) as described [56]. The enzyme activity was calibrated against the protein content of the lysate. The respective values were always considered as to represent the NF-κB activity.

*In pbMEC*: pbMEC were seeded in 6 well plates and transfected with 40 ng/well of the ELAM reporter gene using Lipofectamin 2000 (Invitrogen) as detailed in [9]. The next morning they were stimulated for 24 h and the luciferase activity was measured, as described above.

*As indicated through IκBα degradation*: Degradation of the NF-κB inhibitor IκBα was visualized in Western blots after separating 30 μg of total protein on 12 % SDS PAGE gels (BioRad). The IκBα specific primary antibody was from Santa Cruz (sc-371; diluted 1:1000) and was detected with the HRP conjugated secondary goat-anti rabbit IgG from Cell Signaling (#7074; diluted 1:20000). Blots were developed with the Clarity Western ECL Substrate (BioRad #170-5060) and analyzed on a ChemiDoc Station (BioRad).

### Statistical analysis and data display

The data were analysed with GraphPad Prism Version 5 (GraphPad Software, Inc., La Jolla, CA, USA). Student’s t-test and Bonferroni’s correction for pairwise multiple comparisons was always applied. Heat maps of gene expression were established with Expander 6.0 software (EXPression ANalyzer and DisplayER; [59]). The expression values were standardized by mean 0 and variance 1 to visualize the expression pattern on the same scale.

## Additional files

Additional file 1: Figure S1. Titration of ligand dose for TLR2 activation. **Figure S2.** *TNF* expression recorded in two separate challenge experiments. **Figure S3.** Base line expression of immune genes in pbMEC, 24 h after changing the growth medium from SM10 to either SM Milk or SM0. **Figure S4.** *S. aureus* strain specific difference in the immune response of pbMEC could not be observed in SM10. **Figure S5. A)** Effect of supplementation of SM10 with raw milk or pasteurized milk upon immune gene expression. **B)** Powdered milk closely resembles commercially available UHT milk. **Figure S6.** Comparison of the stability of various housekeeping genes as reference genes for normalization of RT-qPCR data and CLIC1 under various experimental conditions. (PDF 363 kb) Additional file 2: Table S1A. Extent and kinetics of modulated mRNA concentrations after stimulating pbMEC with *E. coli*_1303_ or *S. aureus*_1027_ in different media and for various times. **Table S1B.** Extent and kinetics of modulated mRNA concentrations after stimulating pbMEC with mimetics for lipopeptides or LPS. **Table S2.** List of the oligonucleotide primers used for RT-qPCR quantification. (PDF 187 kb)

## Abbreviations

LPS: Lipopolysaccharide
LTA: Lipoteichoic acid
NF-κB: Nuclear factor of kappa light chain gene enhancer in B cells
MAP-kinases: Mitogen-activated protein kinases
NOD-receptor: Nucleotide-binding oligomerization domain receptor
PAMP: Pathogen associated molecular pattern
RLU: Relative light units
RT-qPCR: Real time quantitative polymerase chain reaction
TLR: Toll-like-receptor
