# Supplementary material for: Extracellular milieu grossly alters pathogen-specific immune response of mammary epithelial cells
Source: BMC Vet Res. 2015 Jul 30;11:172. doi: 10.1186/s12917-015-0489-3 (PMC4518681; doi:10.1186/s12917-015-0489-3)
Supplement: Additional file 1: Figure S1. — Titration of ligand dose for TLR2 activation. Figure S2. TNF expression recorded in two separate challenge experiments. Figure S3. Base line expression of immune genes in pbMEC, 24 h after changing the growth medium from SM10 to either SM Milk or SM0. Figure S4. S. aureus strain specific difference in the immune response of pbMEC could not be observed in SM10. Figure S5. A) Effect of supplementation of SM10 with raw milk or pasteurized milk upon immune gene expression. B) Powdered milk closely resembles commercially available UHT milk. Figure S6. Comparison of the stability of various housekeeping genes as reference genes for normalization of RT-qPCR data and CLIC1 under various experimental conditions. (PDF 363 kb) [file 12917_2015_489_MOESM1_ESM.pdf]

Additional file 1, Supplementary figure S1– Titration of ligand dose for TLR2 activation.
A) *Titration of increasing concentrations of heat killed* E. coli$_{1303}$ *or* S. aureus$_{1027}$ *preparations (abscissa) upon luciferase activity (ordinate) after 24 h.* Values were normalized against the protein concentration within the extracts and are expressed as multiples of the unstimulated controls. All samples were assayed in triplicate. All values from samples stimulated with 3µg/ml or more of the pathogen proteins were statistically significantly ($p<0.05$ from the respective controls; t-test). Methods: HEK 293 cells were transfected with 100 ng of a vector expressing the bovine TLR2 receptor and 40 ng of the NF-κB driven reporter gene construct expressing the Renilla-luciferase. **B)** *Same as before, but the cultures were challenged with the respective di- or tri-acetylated synthetic lipopeptides (Pam2CSK4 or Pam3CSK4).* Different superscript letter indicate significant ($p<0.05$) difference from each other and from the unstimulated control.

Additional file 1, Supplementary figure S2 – *TNF* expression recorded in two separate challenge experiments.
TNF expression (ordinate; relative copy number of cDNA) was recorded at the times indicated (abscissa) after challenging pbMEC with 30 µg/ml of the bacterial proteins in the respective media, as indicated. Values are mean values (± SEM) from duplicate determinations.

Additional file 1, Supplementary figure S3: – Base line expression of immune genes in pbMEC, 24 h after changing the growth medium from SM10 to either SM Milk or SM0.
Relative cDNA copy numbers of the respective genes are expressed as multiples of respective value from controls kept constantly in SM10. Mean values ± SEM from duplicate samples.

Additional file 1, Supplementary figure S4 – *S. aureus* strain specific difference in the immune response of pbMEC could not be observed in SM10.
A) *Genetically distinct strains from* S. aureus (cf [29]) *uniformly induce only low levels of immune gene expression*. Ordinate: relative cDNA copy numbers, expressed as multiples of unstimulated controls; abscissa: time after stimulation. Methods: pbMEC were challenged in

SM10 with 30 µg/ml of protein from heat killed preparations of either *E. coli*$_{1303}$ (caret) or diverse *S. aureus* strains, as indicated. Moreover, co-culturing pbMEC with live *S. aureus*$_{1027}$ (MOI 30) for 1 h followed by killing the bacteria with 100 µg/ml gentamycin and continued culturing of the cells for the times as indicated did not enforce the extent of immune gene induction (red graph). All pathogen preparations were taken from the stationary phase of culture growth. Similar data were recorded using preparations of the same strains taken from the logarithmic phase of culture growth. Also, no strain specific differences could be recorded challenging these cells in SM10 with various dilutions of supernatants from those strains (dilutions 1+2; 1+4 or 1+ 9 volumes of SM10, respectively). **B)** E. coli *but neither of the* S. aureus *strains nor live* S. aureus *pathogens significantly activated NF-κB factors in pbMEC.* **B1)** pbMEC were transfected with 40 ng of the NF-κB reporter gene construct and stimulated for 24 h with 30 µg/ml of proteins from the respective pathogen preparation. **B2)** similar as B1, but the pbMEC were co-cultured for 1 h with live *E. coli*$_{1303}$ or *S. aureus*$_{1027}$ for 1 h. Subsequently the bacteria were killed with gentamycin (100 µg/ml) and the cells were incubated for another 23 h. Values are representative for two biological replica experiments each assayed in triplicate.

Additional file 1, Supplementary figure S5 – **A)** *Effect of supplementation of SM10 with raw milk or pasteurized milk upon immune gene expression*. pbMEC were cultured in SM10 and supplemented with various volumes (0.1; 1; 3% v/v) of reconstituted raw milk or pasteurized milk (72°C, 15 sec followed by spray cooling) from healthy HF cows (control). The cells were eventually stimulated for 3 h with 30 µg/ml of heat killed *E. coli*$_{1303}$ proteins (*E. coli*) and the concentration of mRNA-encoding TNF-α or LAP was measured with RT-qPCR. **B)** *Powdered milk closely resembles commercially available UHT milk.* 30 µg/slot of either UHT-milk (UHT, obtained from the grocery) or dissolved milk powder (MP), as used as supplement in the current

study were resolved on 12% SDS-gels and stained with Coomassie Brilliant Blue. Position of the molecular weight markers is indicated.

Additional file 1, Supplementary figure S6 – Comparison of the stability of various housekeeping genes as reference genes for normalization of RT-qPCR data and CLIC1 under various experimental conditions.

A) pbMEC cultures derived from three different cows (animals 1-3) were challenged in SM10 with 30 µg/ml of heat killed *E. coli*$_{1303}$ proteins for the times as indicated (abscissa). The fold increase over the mRNA concentration recorded at t0 was indicated for CXCL8 (as marker for successful induction) and of CLIC1 (chloride intracellular channel protein 1), RPL35A (ribosomal protein L35a), ACTB (β-actin, cytoskeletal protein) and GAPDH (glyceraldehyde 3-phosphate dehydrogenase). Numbers below the gene symbols are the values of the stability index, as calculated with the NormFinder software. **B)** Stability of *CLIC1* expression under the various challenge conditions of the current study. Relative copy numbers of CLIC1 (ordinate) are given from one of the two replica experiments on which the data from the main study are based.

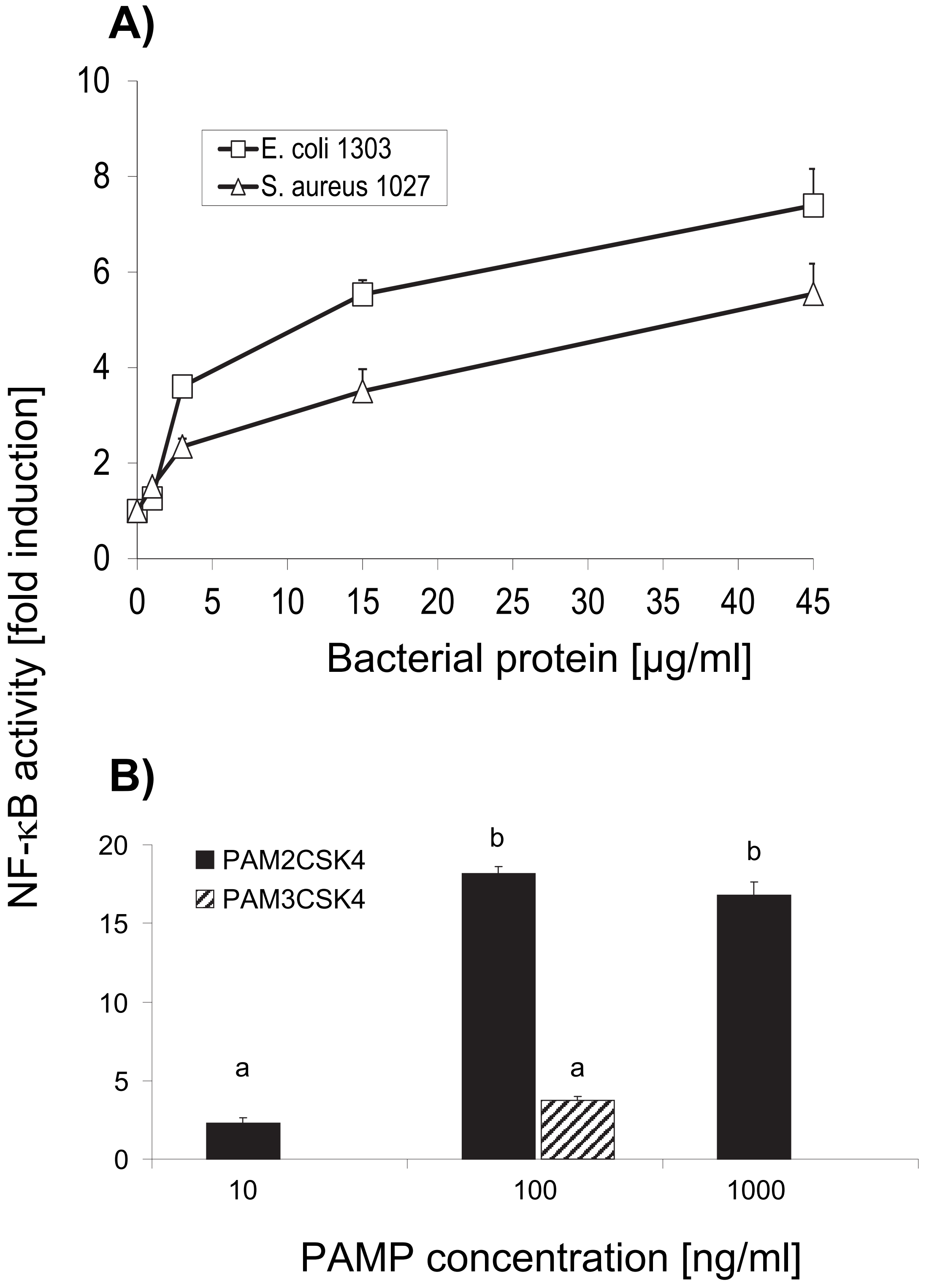

A)
E. coli 1303
S. aureus 1027
NF-κB activity [fold induction]
Bacterial protein [µg/ml]
B)
PAM2CSK4
PAM3CSK4
a
b
a
b
10
100
1000
PAMP concentration [ng/ml]

Figure S1

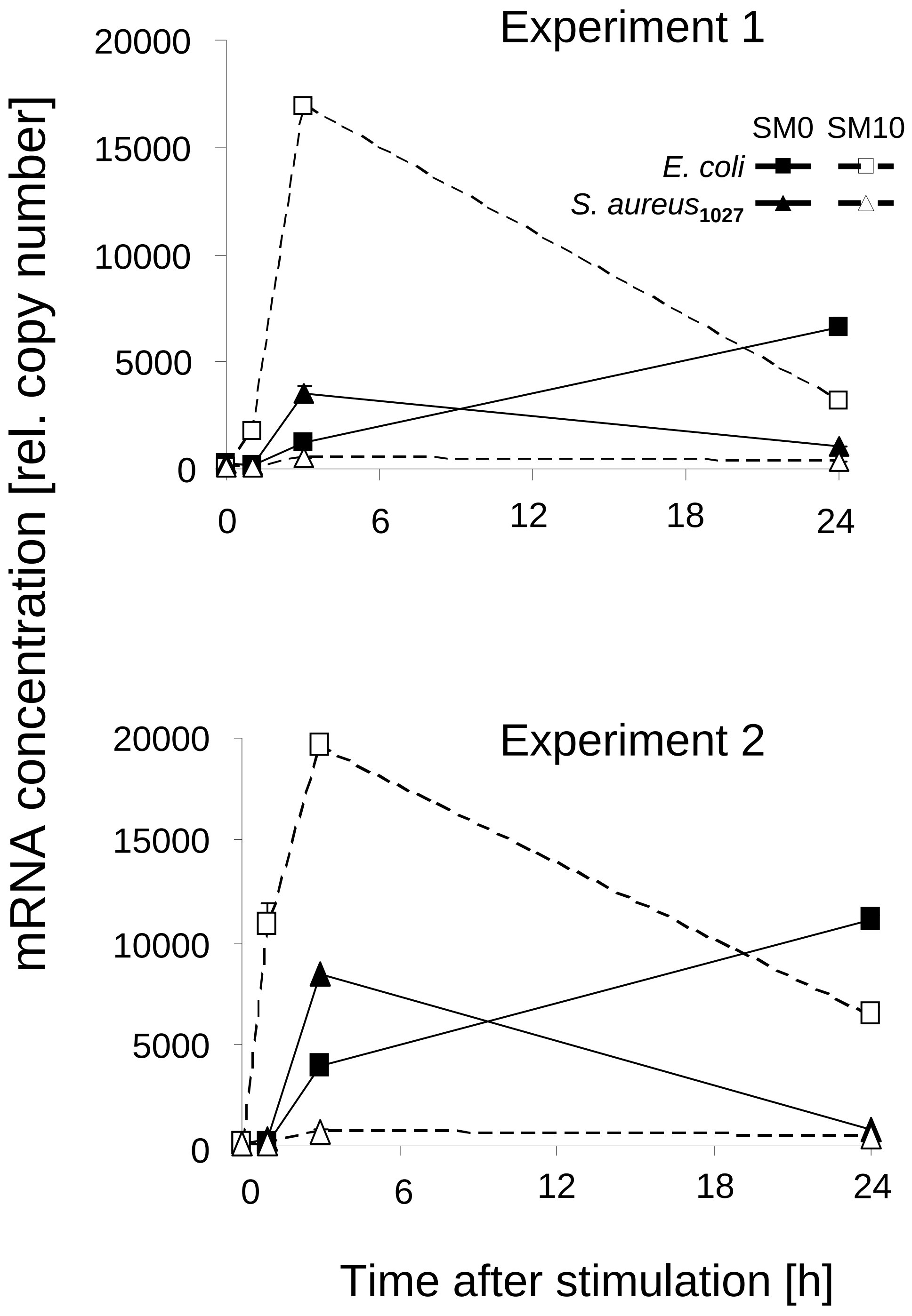
Experiment 1
20000
15000
10000
5000
0
0
6
12
18
24
SM0
SM10
E. coli
S. aureus1027
Experiment 2
20000
15000
10000
5000
0
0
6
12
18
24
mRNA concentration [rel. copy number]
Time after stimulation [h]

Figure S2

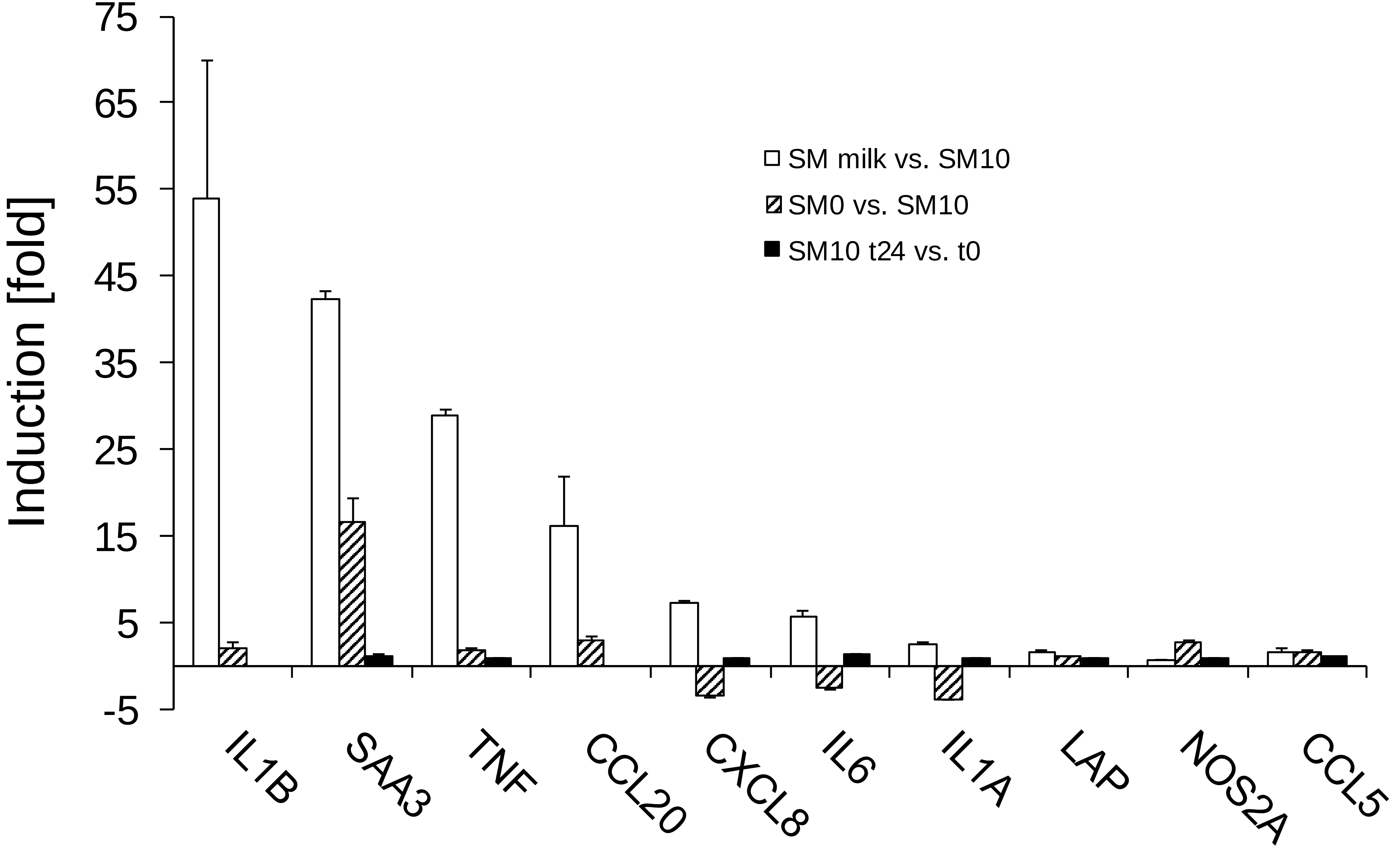
Induction [fold]
75
65
55
45
35
25
15
5
-5
SM milk vs. SM10
SM0 vs. SM10
SM10 t24 vs. t0
IL1B
SAA3
TNF
CCL20
CXCL8
IL6
IL1A
LAP
NOS2A
CCL5

Figure S3

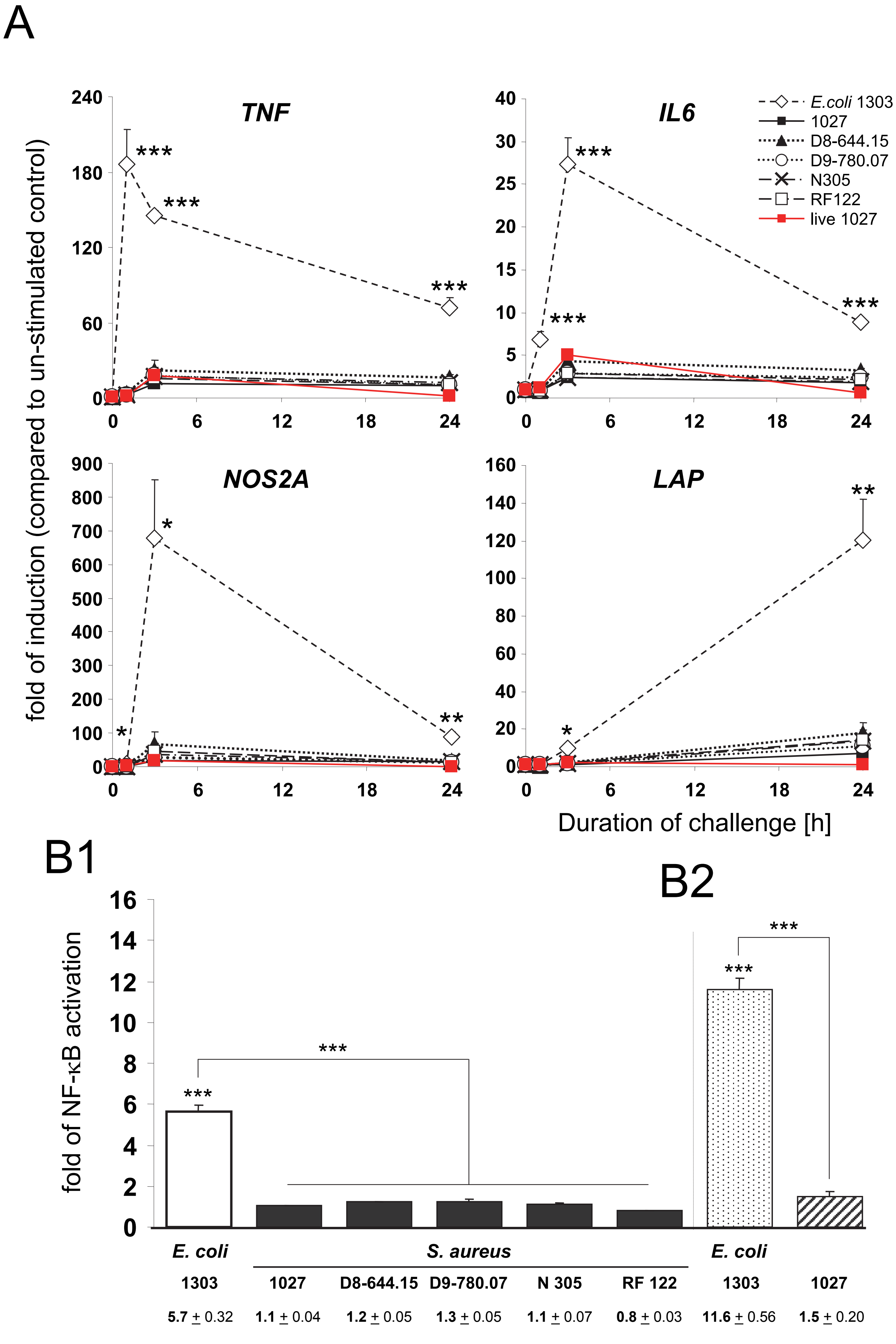
A
TNF
IL6
NOS2A
LAP
fold of induction (compared to un-stimulated control)
Duration of challenge [h]
E.coli 1303
1027
D8-644.15
D9-780.07
N305
RF122
live 1027
B1
B2
fold of NF-κB activation
E. coli
S. aureus
E. coli
1303
1027
D8-644.15
D9-780.07
N 305
RF 122
1303
1027
5.7 ± 0.32
1.1 ± 0.04
1.2 ± 0.05
1.3 ± 0.05
1.1 ± 0.07
0.8 ± 0.03
11.6 ± 0.56
1.5 ± 0.20

Figure S4

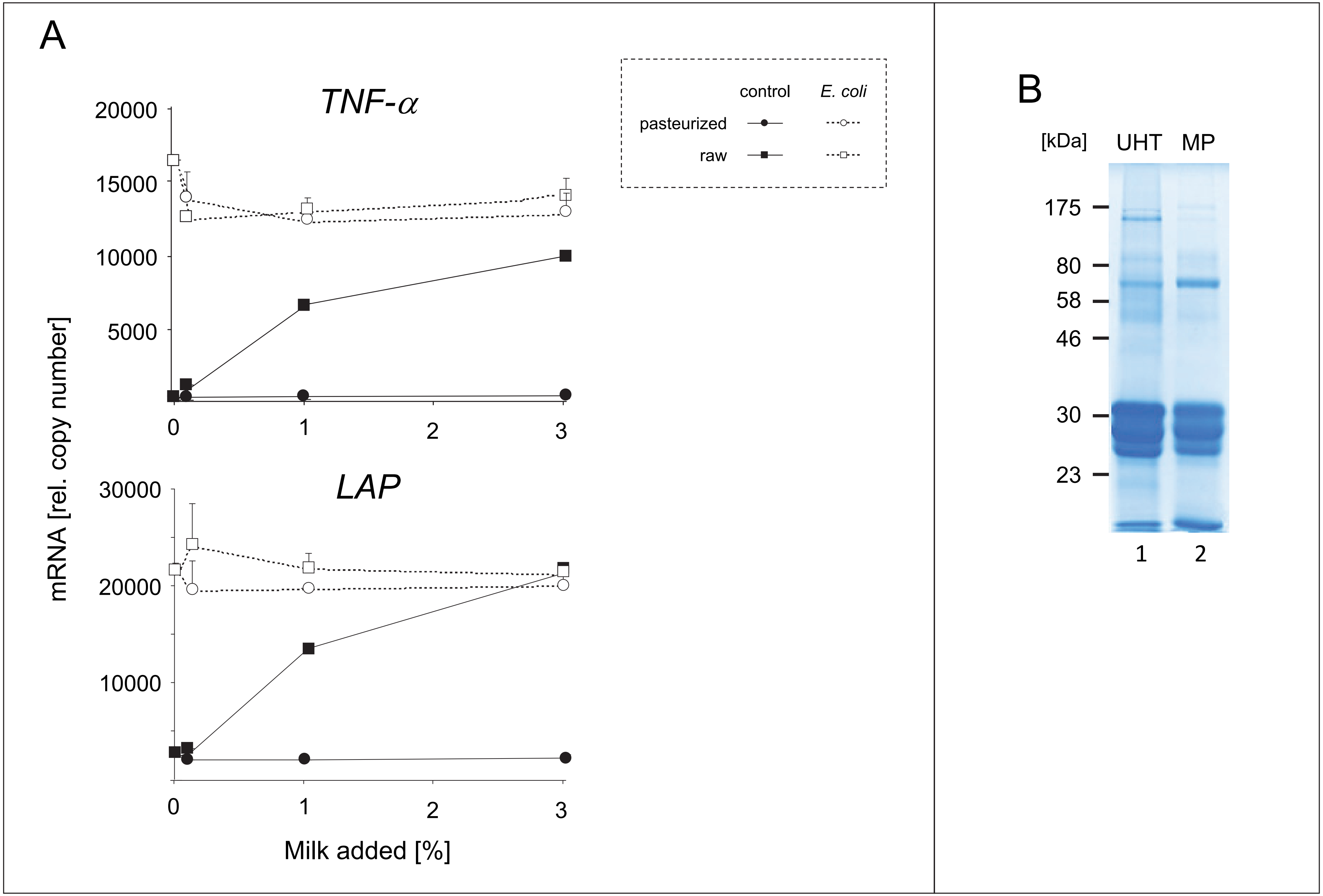
A
TNF-α
LAP
mRNA [rel. copy number]
20000
15000
10000
5000
30000
20000
10000
0
1
2
3
Milk added [%]
control
E. coli
pasteurized
raw
B
[kDa]
UHT
MP
175
80
58
46
30
23
1
2

Figure S5

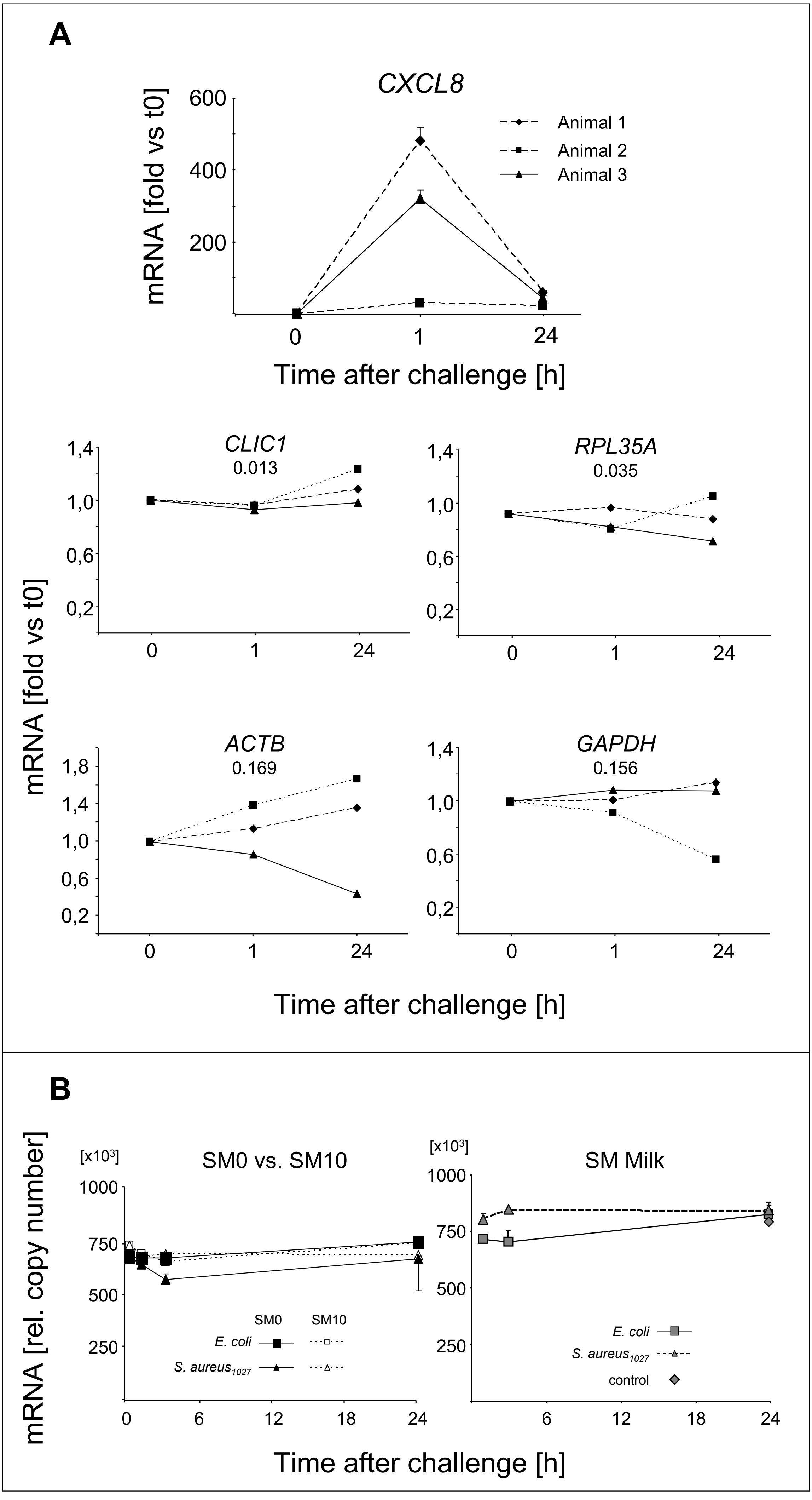

Supplementary Figure S6
