# Supplementary material for: Extracellular milieu grossly alters pathogen-specific immune response of mammary epithelial cells
Source: BMC Vet Res. 2015 Jul 30;11:172. doi: 10.1186/s12917-015-0489-3 (PMC4518681; doi:10.1186/s12917-015-0489-3)
Supplement: Additional file 2: Table S1A. — Extent and kinetics of modulated mRNA concentrations after stimulating pbMEC with E. coli 1303 or S. aureus 1027 in different media and for various times. Table S1B. Extent and kinetics of modulated mRNA concentrations after stimulating pbMEC with mimetics for lipopeptides or LPS. Table S2. List of the oligonucleotide primers used for RT-qPCR quantification. (PDF 187 kb) [file 12917_2015_489_MOESM2_ESM.pdf]

Additional file 2:
- Supplementary table S1A: Extent and kinetics of modulated mRNA concentrations after stimulating pbMEC with *E. coli*$_{1303}$ or *S. aureus*$_{1027}$ in different media and for various times. Values are means (from two biological replica experiments, each assayed in duplicate) ± SEM of fold change relative to unstimulated control; bold face numbers, maximum values for each condition; underlined, $p < 0.05$ vs. unstimulated control. Bonferroni's correction for multiples analyses was applied.

- Supplementary table S1B: - Extent and kinetics of modulated mRNA concentrations after stimulating pbMEC with mimetics for lipopetides or LPS. Values are means (from two biological replica experiments, each assayed in duplicate) ± SEM of fold change relative to unstimulated control; bold face numbers, maximum values for each condition; underlined, $p < 0.05$ vs. unstimulated control. Bonferroni's correction for multiples analyses was applied.

Additional file 2: Supplementary table S2: List of the oligonucleotide primers used for RT-qPCR quantification.

Supplementary table S1A: Extent and kinetics of modulated mRNA concentrations after stimulating pbMEC with *E. coli* strain 1303 or *S. aureus* strain 1027 in different media and at various times.

			E. coli			*S. aureus*$_{1027}$		
**Group**	**Gene**	**Time [h]**	**SM0**	**SM10**	**SM Milk**	**SM0**	**SM10**	**SM Milk**
A	TNF	1	0.9[§] ± *0.04*	64.0 ± *26.3*	1.2 ± *0.1*	1.5 ± *0.3*	1.5 ± *0.4*	1.5 ± *0.4*
		3	14.4 ± *4.1*	**189** ± *4.4*	**22.3** ± *4.4*	**33.5** ± *7.4*	**6.3** ± *0.7*	**8.8** ± *2.9*
		24	**50.2** ± *6.5*	49.6 ± *9.0*	8.1 ± *0.7*	5.4 ± *0.3*	4.2 ± *0.3*	1.7 ± *0.2*
	IL6	1	1.0 ± *0.04*	5.3 ± *2.8*	0.9 ± *0.1*	1.3 ± *0.2*	0.8 ± *0.02*	0.7 ± *0.02*
		3	3.6 ± *0.2*	**25.3** ± *7.6*	3.3 ± *1.2*	**8.8** ± *2.2*	**2.6** ± *0.2*	**1.9** ± *1.0*
		24	**10.7** ± *1.2*	7.4 ± *1.1*	**4.7** ± *0.7*	1.5 ± *0.1*	2.2 ± *0.2*	0.8 ± *0.1*
	IL1A	1	1.5 ± *0.5*	4.0 ± *0.3*	1.4 ± *0.2*	1.4 ± *0.4*	1.1 ± *0.3*	1.1 ± *0.3*
		3	2.9 ± *0.7*	**32.2** ± *4.8*	3.6 ± *0.1*	**6.4** ± *1.8*	**2.3** ± *0.5*	**2.8** ± *0.5*
		24	**8.4** ± *1.8*	5.1 ± *1.1*	**5.6** ± *0.8*	2.0 ± *0.4*	1.4 ± *0.3*	2.1 ± *0.3*
	IL1B	1	1.2 ± *0.2*	5.1 ± *0.8*	1.4 ± *0.4*	0.9 ± *0.2*	1.6 ± *0.5*	1.1 ± *0.2*
		3	20.7 ± *5.2*	**1069** ± *58.4*	24.4 ± *3.5*	**83.7** ± *17.0*	**10.3** ± *0.5*	**5.4** ± *0.3*
		24	**368** ± *15.5*	204 ± *7.1*	**26.8** ± *3.5*	8.0 ± *0.8*	5.2 ± *0.7*	1.1 ± *0.2*
	CXCL8	1	0.6 ± *0.05*	27.3 ± *9.4*	0.9 ± *0.2*	0.7 ± *0.03*	0.6 ± *0.1*	0.9 ± *0.4*
		3	10.8 ± *1.7*	**173** ± *21.7*	**11.6** ± *0.9*	**27.1** ± *5.7*	**7.7** ± *1.5*	**5.2** ± *0.7*
		24	**55.5** ± *1.2*	38.9 ± *7.5*	10.4 ± *1.5*	5.1 ± *0.5*	3.3 ± *0.7*	1.6 ± *0.1*
	CCL20	1	0.6 ± *0.1*	45.4 ± *21.7*	2.0 ± *0.3*	1.0 ± *0.3*	0.4 ± *0.1*	1.9 ± *0.9*
		3	27.1 ± *11.3*	**753** ± *169*	**35.2** ± *4.7*	**129** ± *49.2*	**8.3** ± *2.7*	**16.1** ± *1.5*
		24	**162** ± *24.6*	86.7 ± *21.7*	20.8 ± *2.7*	10.8 ± *2.8*	3.7 ± *1.3*	2.3 ± *0.4*
B	NOS2A	1	2.3 ± *0.2*	6.7 ± *2.5*	0.7 ± *0.3*	2.1 ± *0.4*	0.9 ± *0.1*	0.6 ± *0.3*
		3	15.6 ± *6.9*	**228** ± *87.0*	3.4 ± *0.6*	**62.8** ± *25.6*	3.9 ± *0.5*	2.1 ± *0.3*
		24	**204** ± *56.1*	64.7 ± *27.1*	**22.5** ± *5.4*	16.3 ± *3.3*	**6.1** ± *1.0*	**2.6** ± *0.7*
	LAP	1	1.2 ± *0.2*	0.8 ± *0.1*	0.7 ± *0.1*	1.1 ± *0.1*	0.8 ± *0.1*	0.7 ± *0.2*
		3	1.4 ± *0.1*	4.8 ± *0.7*	0.8 ± *0.1*	2.2 ± *0.1*	1.0 ± *0.05*	1.0 ± *0.3*
		24	**41.8** ± *6.3*	**51.6** ± *11.5*	**6.7** ± *1.4*	**11.4** ± *0.1*	**2.7** ± *0.2*	1.1 ± *0.4*
	SAA3	1	0.5 ± *0.1*	1.8 ± *0.7*	1.5 ± *0.2*	0.5 ± *0.1*	0.5 ± *0.1*	1.2 ± *0.4*
		3	2.5 ± *0.1*	38.1 ± *12.5*	4.0 ± *1.1*	7.0 ± *2.3*	1.6 ± *0.4*	2.3 ± *0.6*
		24	**260** ± *81.4*	**94.2** ± *35.6*	**15.2** ± *2.0*	**28.3** ± *10.5*	**7.6** ± *2.0*	**2.6** ± *0.3*

§ Mean (from two biological replica experiments, each assayed in duplicate) ± SEM of fold change relative to unstimulated control; bold face numbers, maximum values for each condition; underlined, $p < 0.05$ vs. unstimulated control. Bonferroni's correction for multiples analyses was applied.

Supplementary table S1B: Extent and kinetics of modulated mRNA concentrations after stimulating pbMEC with synthetic lipopeptide analogues or LPS.

			**Pam2CSK4**		**Pam3CSK4**		**LPS**	
**Group**	**Gene**	**Time [h]**	**SM0**	**SM10**	**SM0**	**SM10**	**SM0**	**SM10**
A	TNF	1	**247**[§] ± *46*	**230** ± *107*	**101** ± *6*	**147** ± *12*	0.7 ± *0.04*	**138** ± *14*
		3	27.2 ± *5.3*	53.8 ± *3.9*	58.4 ± *12.0*	46.8 ± *3.0*	18.3 ± *2.2*	121 ± *5*
		24	6.8 ± *0.8*	8.1 ± *0.9*	9.9 ± *1.4*	9.4 ± *0.6*	**23.6** ± *3.5*	2.3 ± *0.7*
	IL6	1	**14.0** ± *5.3*	**7.9** ± *2.4*	6.1 ± *0.6*	3.5 ± *0.4*	0.7 ± *0.03*	4.2 ± *0.2*
		3	5.7 ± *3.1*	5.8 ± *0.2*	**11.7** ± *5.8*	**4.2** ± *0.2*	2.9 ± *0.15*	**11.5** ± *0.1*
		24	5.5 ± *0.3*	1.9 ± *0.4*	4.3 ± *1.5*	1.9 ± *0.2*	**5.8** ± *0.2*	0.9 ± *0.0*
	CXCL8	1	**124** ± *32*	**43.6** ± *12.6*	**43.5** ± *8.3*	**22.0** ± *1.0*	1.0 ± *0.02*	**41.3** ± *3.2*
		3	18.2 ± *8.8*	11.7 ± *0.6*	38.0 ± *18.3*	8.9 ± *0.9*	15.1 ± *1.4*	37.5 ± *0.7*
		24	6.2 ±*0.5*	3.3 ± *0.5*	7.5 ± *2.2*	3.8 ± *0.4*	**18.2** ± *1.3*	1.4± *0.1*
B	NOS2A	1	19.3 ± *4.3*	10.3 ± *5.0*	7.4 ± *1.9*	5.0 ± *0.3*	0.4 ± *0.01*	7.9 ± 0.5
		3	**93.5** ± *17.6*	**101** ± *12*	**117** ± *12.8*	**68.6** ± *1.4*	10.8 ± *2.0*	**294** ± *6*
		24	6.4 ± *0.5*	3.4 ± *0.6*	9.5 ± *0.2*	6.4 ± *0.6*	**52.1** ± 9.7	1.5 ± *0.1*

§ Mean (from 2 experiments, each assayed in duplicate) ± SEM of fold change relative to unstimulated control; nd, not determined; bold face numbers, maximum values for each condition; underlined, $p < 0.05$ vs. unstimulated control. Bonferroni's correction for multiples analyses was applied.

Supplementary table S2: Sequences of the oligonucleotide primers used for real-time PCR quantification.

Gene	GenBank no.	Primer sequence (5´–3´)	Amplificate size (bp)
TNF	NM_173966.2	CTTCTGCCTGCTGCACTTCG *<br>GAGTTGATGTCGGCTACAACG *	156
IL6	NM_000600.3	GGAGGAAAAGGACGGATGCT<br>GGTCAGTGTTTGTGGCTGGA	227
CXCL8	NM_173925	CCTCTTGTTCAATATGACTTCCA<br>GGCCCACTCTCAATAACTCTC	170
IL1A	NM_174092	GGCCAAAGTCCCTGACCTCT<br>CTGCCACCATCACCACATTC	224
IL1B	NM_174093.1	AACCGAGAAGTGGTGTTCTGC<br>TTGGGGTAGACTTTGGGGTCT	167
CCL20	NM_174263.2	CAGCAAGTCAGAAGCAAGCAA<br>CCCACTTCTTCTTTGGATCTGC	179
NOS2A	NM_001076799	ACAGGATGACCCCAAACGTC<br>TCTGGTGAAGCGTGTCTTGG	188
SAA3	NM_181016.3	CTTTCCACGGGCATCATTTT<br>CTTCGGGCAGCGTCATAGTT	188
LAP	NM_203435	AGGCTCCATCACCTGCTCCTT<br>CCTGCAGCATTTTACTTGGGCT	182
TTP	NM_174493.1	CATCCTTCGGCCATCTGC<br>CTGGAGTCGGATGAGCTGAG	155
CLIC	NM_001015608	GTCTCAGTCCGCCTCTTGGT<br>AGAACAACCGCAGGTCGAAT	153

*upper line: forward-, lower line, reverse-primer
